# 

**Published:** 1858-12

**Authors:** 


### CONTENTS.

ORIGINAL COMMUNICATIONS— PAGE.

A Public Address on the Advancement of Medical Science. By O. Q. Herrick, M.D.... 613

Power of "Accommodation" in the Human Eye; its Physiology and Pathology. By E. L. Holmes, M.D.... 623

Resection of the Elbow Joint and Ulna; recovery, with a useful member. By J. W Freer, M.D.... 632

Diabetes. By W. Matthews, M.D.... 635

Report of Committee on Practical Medicine. (Continued).... 645

EDITORIAL—

Close of the Volume.... 662

Personal.... 662

Summary of Clinical Cases presented to the Medical Class attending the Mercy Hospital during the Month of November, 1858. By E. O. F. Roler.... 662

In Memoriam.... 665

EXTRACTS—

Trousseau's Opinion of Tracheotomy.... 666

Death from Chloroform.... 667

A Specific for Scabies.... 667

Cold Applications and Sulph. of Copper in Croup.... 667

Strychnine Poisoning Relieved with Camphor.... 668

